# Autoimmune Encephalitis with Neuronal Surface Autoantibodies and Other Suspected Cases of Autoimmune Etiology: A Single-Center Experience in Poland

**DOI:** 10.3390/ijms26199541

**Published:** 2025-09-30

**Authors:** Iwona Kurkowska-Jastrzębska, Katarzyna Polanowska, Katarzyna Kurczych, Agnieszka Cudna, Halina Sienkiewicz-Jarosz, Agnieszka Piechal

**Affiliations:** 1 Second Department of Neurology, Institute of Psychiatry and Neurology, 02-957 Warsaw, Poland; ikurkowska@ipin.edu.pl (I.K.-J.);; 2 First Department of Neurology, Institute of Psychiatry and Neurology, 02-957 Warsaw, Poland; 3 Maria Sklodowska-Curie Medical Academy in Warsaw, 00-136 Warsaw, Poland; 4 Department of Clinical and Experimental Pharmacology, Centre for Preclinical Research and Technology CePT, Medical University of Warsaw, 02-097 Warsaw, Poland

**Keywords:** autoimmune encephalitis, anti-NMDAR, anti-LGI1, anti-CASPR2, limbic encephalitis, differential diagnosis

## Abstract

Autoimmune encephalitis (AE) is an autoantibody-mediated central nervous system disorder with diverse neuropsychiatric and neurological manifestations, and should be considered in the differential diagnosis of acute and subacute neurological or psychiatric syndromes. In this retrospective study, we analyzed 65 patients: 54 with AE (47 antibody-positive, seven antibody-negative) and 11 antibody-positive without AE. The most frequently detected antibodies targeted N-methyl-D-aspartate receptor (NMDAR), leucine-rich glioma-inactivated protein 1 (LGI1), and contactin-associated protein-like 2 (CASPR2)—key synaptic and axonal membrane proteins involved in excitatory neurotransmission, neuronal signaling, and synaptic plasticity. Clinical presentations were heterogeneous, ranging from common neuropsychiatric, cognitive, and seizure manifestations to atypical brainstem or cerebellar features. Symptom distribution analysis further demonstrated distinct patterns among Ab-positive AE, Ab-negative AE, and Ab-positive non-AE groups, with specific symptom–antibody associations providing potential diagnostic clues. Diagnostic complexity was underscored by unusual age at onset, overlap with multiple sclerosis, cases preceded by herpes labialis, and dual-antibody detection. A subset of antibody-positive patients had alternative diagnoses, highlighting the need for careful clinical correlation and cautious interpretation of antibody results. These findings illustrate the diagnostic challenges and broad clinical spectrum of AE, emphasizing the importance of integrating serological, clinical, and imaging data to improve diagnostic accuracy and guide management.

## 1. Introduction

Autoimmune encephalitis (AE) with autoantibodies (Abs) is a heterogeneous group of inflammatory brain disorders associated with post-infectious, paraneoplastic, or idiopathic mechanisms. It is characterized by the presence of pathogenic Abs directed against neuronal surface antigens, including receptors, ion channels, or membrane-associated proteins, involved in essential brain functions. These Abs may impair neurophysiological function through receptor internalization, cross-linking, and functional blockade [1,2], triggering an immune response that, in addition to causing typical signs of encephalitis such as altered consciousness, can lead to a broad spectrum of neurological and neuropsychiatric symptoms, including cognitive and behavioral changes, seizures, abnormal movements, sleep disturbances, and other neurological deficits [3,4,5,6].

Several well-characterized forms of AE involve Abs against synaptic receptors such as the N-methyl-D-aspartate receptor (NMDAR), α-amino-3-hydroxy-5-methyl-4-isoxazolepropionic acid receptor (AMPAR), and γ-aminobutyric acid type A and B receptors (GABA_A_R and GABA_B_R), as well as components of the voltage-gated potassium channel complex, notably leucine-rich glioma-inactivated 1 (LGI1) and contactin-associated protein-like 2 (CASPR2) [6,7,8,9]. Although AE is typically associated with a single Ab, rare cases exhibit coexisting neuronal Abs, a phenomenon that may significantly influence clinical presentation, disease course, and prognosis [10].

Limbic encephalitis (LE) remains the prototypical and most extensively studied AE subtype, defined by the rapid onset of confusion, memory impairment, mood disturbances, and seizures [11,12]. However, increasing evidence indicates that AE can mimic a wide range of neurological, neurodegenerative, and psychiatric syndromes, including first-episode psychosis and new-onset epilepsy [13]. Atypical, milder, or incomplete presentations may also occur, including Ab-negative AE or cases involving Abs of uncertain pathogenic relevance.

The current incidence of Ab-mediated AE is estimated at 13.7 per 100,000, comparable to that of infectious encephalitis [14]. Nevertheless, the number of recognized cases continues to rise, driven by expanding Ab panels, improved molecular diagnostics such as cell-based assays, and growing clinical awareness. Despite these advances, AE remains frequently underdiagnosed or misdiagnosed due to its clinical heterogeneity, nonspecific symptoms, and overlap with inflammatory, neurodegenerative, or psychiatric disorders. Further obstacles include inconsistent access to Ab testing, the absence of standardized early diagnostic protocols, often nonspecific or normal MRI findings, and the persistent misconception of AE as a rare disease.

A more comprehensive understanding of AE’s immunopathogenesis, molecular targets, and clinical spectrum is essential to promote earlier diagnosis and treatment, both of which are critical to improving outcomes in these often disabling but potentially reversible conditions.

In this study, we present a retrospective case series of patients evaluated for AE, including individuals with and without detectable neuronal surface Abs, as well as those with a revised final diagnosis. We describe their clinical and demographic profiles, integrating immunological findings and neuroimaging results. Particular focus is placed on rare and diagnostically challenging cases involving comorbidities, dual Abs, and Ab-positive patients who did not meet diagnostic criteria for AE.

## 2. Results

### 2.1. Demographic and Diagnostic Characteristics of the Study Cohort

Among the 65 patients (35 females, 30 males), 64 were adults (≥18 years) and one was 16 years old (age range 16–84 years; median age 53). Of these, 54 were diagnosed with AE (47 Ab-positive/definite, 72.3%; 7 Ab-negative, 10.8%), while 11 (16.9%) were Ab-positive without meeting AE diagnostic criteria. Patients with Ab-positive AE (median age 61) and Ab-negative AE (median age 55) were generally older than Ab-positive individuals without AE (median age 35).

#### 2.1.1. Autoantibody Profiles and CSF Antibody Testing

Of the 58 patients with detected cell-surface Abs (47 with Ab-positive AE and 11 Ab-positive without AE), most had monospecific Abs. Three AE patients had double Ab positivity: one with two cell-surface Abs (LGI1/NMDAR) and two with coexisting Abs against both cell-surface and intracellular antigens (GABABR/Hu and AMPAR/Hu).

All Abs were detected in serum, except for one NMDAR case in which the Ab was found exclusively in CSF. CSF testing was available for a subset of patients, limited by sample availability and, particularly between 2013 and 2016, by variability in clinical practice regarding the necessity of CSF analysis when serum Abs were already detected.

The distribution of cell-surface Abs among Ab-positive AE patients—listed in descending order of frequency—was: anti-NMDAR (*n* = 24), anti-LGI1 (*n* = 13), anti-CASPR2 (*n* = 8), anti-GABABR (*n* = 2), and anti-AMPAR (*n* = 1). Anti-NMDAR AE occurred predominantly in females, whereas anti-LGI1 and anti-CASPR2 AE were more frequent in males. Most patients were older adults, with the highest median age in the anti-CASPR2 group (70 years), followed by anti-LGI1 (52 years) and anti-NMDAR (51.5 years). Age distribution comparisons among the anti-NMDAR, anti-LGI1, and anti-CASPR2 groups showed no statistically significant differences [Kruskal–Wallis test, H(2, N = 43) = 2.51; *p* = 0.28]. One patient with double NMDAR/LGI1 positivity was excluded from analysis due to uncertain attribution of the clinical presentation. CSF Ab testing was negative in 17 patients and not performed in 15.

In contrast, Ab-positive patients without AE had Abs against either NMDAR (*n* = 7) or CASPR2 (*n* = 4), all detected in serum. The NMDAR subgroup (median age 48 years) was older than the CASPR2 subgroup (median 24.5 years). CSF Ab testing was negative in two cases and not performed in nine. Table 1 summarizes Ab distribution by AE status.

#### 2.1.2. CSF Analysis

CSF examination was performed in 56 patients. No abnormalities were found in 20 cases. Pleocytosis was observed in 14 patients (including three with >50 cells/mm^3^), and elevated protein levels were detected in 18. Oligoclonal bands were present in 19 patients: 12 with type 2 or 3 patterns indicating an intrathecal inflammatory response, six with type 4 patterns suggestive of blood–brain barrier dysfunction and systemic inflammation, and 1 with a monoclonal band (Table 2).

#### 2.1.3. MRI Findings

All 65 patients underwent brain MRI. No abnormalities were detected in 20 patients (30.8%), including 15 with AE (14 Ab-positive AE, 21.5%; 1 Ab-negative AE, 1.5%) and five Ab-positive individuals without AE (7.7%). Typical limbic changes characteristic of encephalitis were observed in 19 patients (29.2%). Among other abnormal findings, the most common were vasogenic lesions (20, 30.8%), followed by demyelination (3, 4.6%), neoplastic lesions (2, 3.1%), and cerebral atrophy (1, 1.5%). MRI findings by Ab status are presented in Table 3.

#### 2.1.4. Neoplasm Associations

All patients were evaluated for underlying neoplasms, and 14 cases were identified overall: 10 (15.4%) among Ab-positive AE patients, 1 (1.5%) in Ab-negative AE, and three (4.6%) among Ab-positive individuals without AE. Table 4 shows neoplasm distribution by Ab status.

#### 2.1.5. Immunosuppressive Treatment

Initial treatment consisted of intravenous methylprednisolone (MP) at a dose of 1 g/day for five consecutive days. In patients who did not respond adequately, second-line therapy included intravenous immunoglobulin (IVIG) at a total dose of 2 g/kg administered over 3–5 days, or plasma exchange (PLEX). Subsequent immunosuppressive treatment included cyclophosphamide (CYC) or rituximab; the latter was used in only one case, reflecting limited prior clinical experience with this agent. Approximately half of the AE patients (28/54) received long-term immunosuppressive therapy, most commonly with oral corticosteroids, mycophenolate mofetil, or azathioprine (Table 5).

Given the 11-year timespan of the study, maintenance immunotherapy was not routinely initiated in earlier cases. In those instances, treatment was typically deferred until clinical relapse or reserved for syndromes with a particularly severe initial presentation. In contrast, during the past 2–3 years, patients have more consistently received long-term maintenance immunosuppression from the outset.

### 2.2. Clinical Presentation and Supportive Investigations

All patients underwent clinical assessments at various stages of the disease. Those who survived hospitalization were subsequently monitored through outpatient follow-up (1–7 years, depending on the case) to track symptoms and guide treatment. The diagnosis of AE was established according to the criteria of Graus et al. [12], with exclusion of other probable diseases (Materials and Methods section).

#### 2.2.1. Findings in AE Patients

Among the 54 patients with AE, clinical symptoms most commonly indicated limbic system involvement (*n* = 41; 75.9%). Less frequent presentations included cortico-subcortical dysfunction presenting as rapidly progressive dementia (RPD; *n* = 6; 11.1%), cerebellar involvement (*n* = 4; 7.4%), and brainstem involvement (*n* = 3; 5.6%).

Initial symptoms were predominantly psychiatric (psychotic or behavioral changes), with cognitive deficits, motor disturbances, seizures, and altered consciousness also common. Speech disturbances emerged as a distinct symptom group and were most frequently observed in patients with anti-NMDAR Abs. Hyponatremia was primarily associated with anti-LGI1 and CASPR2 Abs, while polyneuropathy was noted in a single case of anti-CASPR2 AE. Only four patients (7.4%) achieved complete recovery (three Ab-positive AE, one Ab-negative AE).

CSF was normal in almost half of patients (26/54). Elevated cytosis was found in 13 patients, elevated protein in 12, and CSF was not tested in six. MRI was normal in 15 cases; temporal lobe involvement was found in 19, vasogenic changes in 17, demyelinating lesions typical of MS in two, and atrophy in one. Neoplasms were identified in 11 (20.4%) patients (10 Ab-positive AE, one Ab-negative AE).

Due to the retrospective nature of the study, data on prodromal symptoms were limited. Nevertheless, in half of the cases (*n* = 27), recent potential triggers such as vaccinations, mild upper respiratory tract infections, herpes labialis, or herpetic encephalitis were reported within 2–4 weeks before disease onset.

##### Characteristics of Ab-Positive AE Patients

Anti-NMDAR Encephalitis

Anti-NMDAR AE, including one case with dual anti-LGI1/NMDAR Ab positivity, was the most common subtype (*n* = 24/54; 44.4%), with a female predominance (16 females and eight males; female-to-male ratio 2:1) and a median age of 51.5 years. Most patients presented with an LE phenotype (*n* = 18; 75%), characterized by psychiatric or cognitive symptoms, mutism, and seizures. Less frequent presentations included RPD, cortico-subcortical involvement, and brainstem involvement (each *n* = 2; 8.3%). Tumor screening identified ovarian tumors in two women (a teratoma at age 22 and a carcinoma at age 73) and prior malignancies in two others (renal cell carcinoma, *n* = 1; breast cancer, *n* = 1).

Selected cases illustrate the clinical variability of anti-NMDAR AE. Among patients with the LE phenotype, one was transferred from a psychiatric unit following seizures that occurred during a first psychotic episode. Another female patient was referred after six months of treatment-resistant first-episode psychosis with seizures, without any clinical improvement.

A male patient developed encephalitis approximately four weeks after recurrent herpes labialis and within days of receiving a COVID-19 mRNA vaccine. One week post-vaccination, he experienced bilateral tonic–clonic seizures, followed by memory impairment, mutism, and altered consciousness. He was hospitalized and treated for suspected meningoencephalitis (CSF: 6 cells/mm^3^, protein 35 mg/dL), but viral etiologies were excluded. Anti-NMDAR Abs were detected in serum only (titer 1:1000). Following intravenous MP and IVIG, he made a full recovery and remained asymptomatic and seronegative for over four years. This case suggests that herpes labialis may contribute to the induction of anti-NMDAR autoimmunity.

Two female patients with multiple sclerosis (MS) developed AE independently of their underlying disease. The first (22 years) was diagnosed with both MS and AE after two bilateral tonic–clonic seizures, prolonged impaired consciousness, and speech disturbances. Status epilepticus was excluded, but EEG showed frontal slowing. Despite normal CSF and CT findings, anti-NMDAR Abs were detected in serum and CSF (titer 1:100). She received intravenous MP with clinical improvement. MRI revealed multiple demyelinating lesions in both cerebral hemispheres and the cerebellum. Retrospective history showed episodes of transient limb paresis and paraesthesias over the previous three years, and CSF analysis confirmed type 2 oligoclonal bands. Initially, only levetiracetam was administered due to the patient’s refusal of MS-specific therapy. Later, a brainstem relapse with oculomotor symptoms occurred; treatment with ofatumumab resulted in good clinical response. At three-year follow-up, she remains seizure-free and anti-NMDAR Ab-negative.

The second woman (45 years), with a 20-year history of MS (EDSS score 6/10; four-limb weakness and ataxia), was admitted for non-motor status epilepticus despite no prior epilepsy. Previous treatments included interferon, dimethyl fumarate, and most recently siponimod. She had also been receiving SSRIs and benzodiazepines for over a decade for comorbid depression and anxiety. MRI revealed no new demyelinating lesions, and CSF excluded infection. During hospitalization, she developed acute psychosis (hallucinations, delusions, anxiety) and cognitive decline. Anti-NMDAR Abs were detected in serum and CSF (titers > 1:100). High-dose intravenous MP and IVIG led to marked improvement, although she remained bedridden and required prolonged rehabilitation. Maintenance therapy included siponimod and three additional IVIG cycles (2 g/kg every four weeks), enabling return to baseline neurological status. One year later, she experienced a milder AE relapse with seizure worsening and behavioral symptoms, accompanied by Ab recurrence, and again responded to MP and IVIG. Additional immunomodulatory therapy beyond siponimod is under consideration.

Among rare non-limbic presentations, cortico-subcortical forms of anti-NMDAR AE had a subacute onset of memory impairment, tremor, and bradykinesia, initially mimicking parkinsonian syndromes. One female patient (64 years) also developed urinary incontinence and lower limb paresis, while another male patient (52 years) presented with ataxia. Brainstem presentations included a subacute onset of cerebellar syndrome, limb paresis, and oculomotor disturbances.

Only two patients achieved complete recovery without relapse. Symptom recurrence occurred in five, all responding well to prolonged immunosuppressive treatment. Long-term sequelae included cognitive decline (*n* = 7), persistent psychiatric symptoms (*n* = 4), and epilepsy (*n* = 4). There were eight deaths: carcinoma-related (*n* = 2); progressive multifocal leukoencephalopathy 10 years after AE onset with two relapses and long-term immunosuppression (*n* = 1); complications/disability (*n* = 3); and unknown cause (*n* = 2).

Anti-LGI1 Encephalitis

Anti-LGI1 AE, including the previously mentioned dual anti-LGI1/NMDAR Ab case, was diagnosed in 13/54 AE patients (24.1%), with a male predominance (nine males and four females; male-to-female ratio 2.25:1) and a median age of 52 years.

Symptoms were predominantly limbic, with 11 of 13 patients progressing to classic LE. Two cases presented with RPD; one was a 23-year-old male with dual Abs who, in addition to RPD, experienced seizures, both persisting over time. Hyponatremia and new-onset seizures—most commonly dyscognitive—were frequent initial findings. Seizures occurred in all patients, with faciobrachial dystonic seizures (FBDS) reported in two cases.

Two patients had neoplastic associations: one with a nasopharyngeal tumor diagnosed during treatment and one with a history of melanoma diagnosed ten years earlier. No new malignancies were detected during follow-up.

Overall outcomes were suboptimal. Only one patient achieved full recovery; others developed epilepsy (*n* = 8), cognitive decline (*n* = 6), and persistent psychiatric symptoms (*n* = 4). Two patients died: one with a tumor, and one with fulminant disease—featuring refractory status epilepticus and respiratory failure—associated with a high serum Ab titer (1:10,000), who developed cardiac arrest during intensive care unit treatment despite four plasmapheresis courses.

Anti-CASPR2 Encephalitis

Anti-CASPR2 AE was diagnosed in 8/54 AE patients (14.8%; five males and three females), with a median age of 70 years. They most commonly presented with LE (*n* = 5), followed by RPD (*n* = 2) and slowly progressive cerebellitis (*n* = 1). No cases of myokymia–a typical CASPR2-related feature–were observed. No neoplasms were identified in this subgroup.

In patients with LE, the clinical picture included memory loss, seizures, and behavioral changes. One patient also presented with chronic polyneuropathy, likely unrelated to AE.

The two men with RPD experienced gradual memory decline over a 7-month period. One was hospitalized for seizures, while the other was referred for evaluation due to cognitive and behavioral symptoms accompanied by a parkinsonian syndrome.

One additional male patient presented with a progressive cerebellar syndrome that had begun with a relatively acute onset seven years earlier. Initial CSF analysis showed lymphocytic pleocytosis (20–30 cells/mm^3^), elevated protein, and type 3 oligoclonal bands. Multiple tests for onconeural and GAD65 Abs were negative. MRI demonstrated cerebellar atrophy without signs of inflammation. Treatment with MP and IVIG slowed the progression of ataxia but did not achieve full recovery. Several years later, the patient’s condition worsened; re-evaluation revealed anti-CASPR2 Abs and CSF abnormalities similar to those previously observed. MP followed by oral prednisone stabilized his cerebellar syndrome

Anti-GABA_B_R Encephalitis

Anti-GABA_B_R AE was diagnosed in 2/54 AE patients (3.7%; both females in their sixth decade of life): one with brainstem involvement and the other with cerebellar involvement.

The first woman (67 years) presented with a two-month history of progressive gait instability, unintentional weight loss, and fatigue. Electromyography was consistent with Lambert–Eaton syndrome. Serum tested positive for both anti-GABA_B_R and anti-Hu Abs. Chest CT revealed a pulmonary mass, subsequently diagnosed as small-cell lung carcinoma (SCLC), which proved fatal.

The second woman (65 years) presented with a five-month history of subacutely progressive cerebellar symptoms, including gait ataxia, dysarthria, dysphagia, urinary hesitancy, and right-sided ptosis with convergent strabismus. Brain MRI revealed multiple T2-hyperintense lesions in the subcortical and periventricular regions, as well as in the right middle cerebellar peduncle; one lesion demonstrated contrast enhancement. CSF analysis showed type 3 oligoclonal bands, and both serum and CSF tested positive for anti-GABA_B_R Abs. The patient responded well to intravenous MP, with improvement in balance and ataxia. On follow-up, serum Abs were no longer detectable, however, new contrast-enhancing brain lesions appeared, leading to an additional diagnosis of primary progressive MS.

Anti-AMPAR Encephalitis

Anti-AMPAR AE was diagnosed in 1/54 AE patients (1.9%; female, 65 years) with dual anti-AMPAR/Hu Ab positivity. She developed RPD during treatment with atezolizumab for non-small cell lung cancer. Despite immunotherapy, she died 10 months after AE diagnosis due to encephalitis with severe cognitive and pyramidal deficits. No evidence of active malignancy was found at the time of death.

##### Characteristics of Ab-Negative AE Patients

Ab-negative AE was diagnosed in 7/54 AE patients (13.0%; 3 females and 4 males) with a median age of 55 years. With the exception of one patient, six presented with clinical features of LE. Among these, five showed typical limbic changes on MRI, while one demonstrated vasogenic edema. CSF was normal in one case, showed pleocytosis in two, elevated protein in two, and was not tested in one.

Overall outcomes were suboptimal: three patients developed persistent symptoms—cognitive decline (*n* = 2), psychiatric manifestations (*n* = 1), and epilepsy (*n* = 1)—while three others died. The only patient who achieved full recovery was a 26-year-old woman with both limbic and extralimbic features (altered consciousness, psychosis, dysarthria, and signs of brainstem and cerebellar involvement), a normal MRI, and a positive tumor screening (ovarian teratoma), suggesting possible anti-NMDAR AE with a false-negative antibody result.

##### Symptom Patterns and Similarities in AE Patients

**Figure 1:**
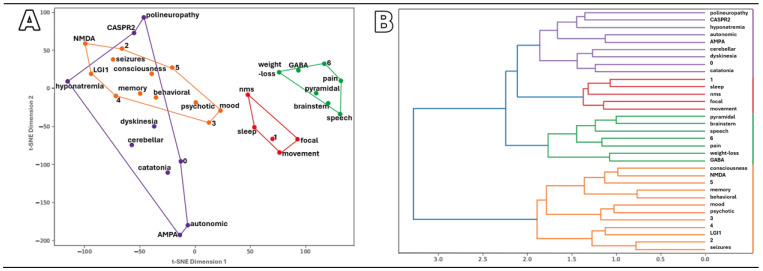

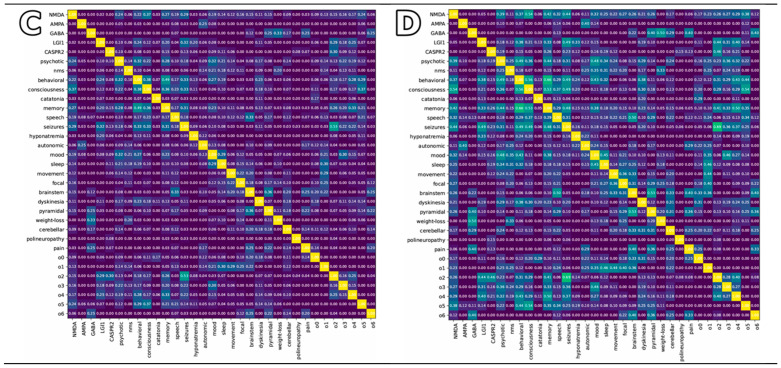
Visualization of relationships between clinical symptoms, Abs, and outcomes: (**A**) t-SNE plot (Jaccard dissimilarity), (**B**) hierarchical plot, (**C**) heat map (Jaccard), and (**D**) heat map (Dice). NMDA, LGI1, CASPR2, AMPA, and GABA indicate the Abs identified; symptom names are given as specific disorders (e.g., polyneuropathy, dyskinesia, nms–neuroleptic malignant syndrome) or as categories (e.g., “memory” for memory disorders). Outcomes at 12 months after AE diagnosis are coded as: 0—no symptoms; 1—relapse; 2—epilepsy; 3—psychiatric disorder; 4—cognitive decline; 5—death; 6—other focal symptoms. Colors indicate clusters from hierarchical clustering of the same dissimilarity matrices.

Despite small numbers in some AE subgroups, clear and interpretable patterns emerged. Patients with anti-NMDAR and anti-LGI1 Abs consistently showed a LE profile—the most common presentation in our cohort—regardless of the clustering method. In contrast, the observed association between anti-CASPR2 Abs and either polyneuropathy or hyponatremia came from a single case with prominent peripheral nerve symptoms, prompting hospital referral and electromyography. Hyponatremia (serum sodium < 135 mEq/L) was rare in both anti-LGI1 and anti-CASPR2 AE. Overall similarity patterns are shown in Figure 1.

#### 2.2.2. Findings in Ab-Positive Patients Without AE

In 11 Ab-positive patients not meeting AE criteria (16.9% of the cohort), Abs most frequently targeted NMDAR (*n* = 7; three females and four males) and less commonly CASPR2 (*n* = 4; three females and one male), all detected in serum. CSF antibody testing was negative in two cases and not performed in nine. CSF analysis was normal in six patients and showed elevated protein in two; it was not performed in three. MRI was unremarkable in five cases, while abnormal findings included glioma (*n* = 2), stroke (*n* = 1), nonspecific small hyperintensities likely of ischemic origin (*n* = 2), and demyelinating lesions characteristic of multiple sclerosis (*n* = 1).

**Figure 2:**
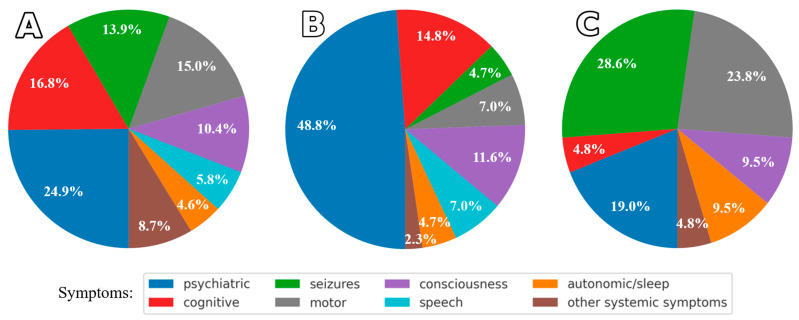
Symptoms in (**A**) patients with Ab-positive AE, (**B**) patients with Ab-negative AE, and (**C**) Ab-positive individuals without AE. Colors represent: psychiatric (behavioral, psychotic, anxiety, depression, bipolar disorders, neuroleptic malignant syndrome, catatonia), cognitive (memory disorders, dementia), seizures (seizures, status epilepticus, myoclonus), motor (parkinsonism, dyskinesia, PSP-like, lower limb paresis, pyramidal syndrome, focal symptoms, cerebellar syndrome, oculomotor symptoms), consciousness (altered consciousness), speech (dysarthria, aphonia), autonomic/sleep, and other systemic symptoms (pain, paresthesia, polyneuropathy, hyponatremia, weight loss, dysphagia, urinary symptoms).

At the time of Ab testing, all patients were diagnosed with AE based on symptoms, most commonly seizures, psychiatric manifestations, and motor disturbances (Figure 2). Reasons for Ab testing included exacerbation of preexisting symptoms, as in epilepsy (in two anti-NMDAR cases, previously diagnosed epilepsy was accompanied by increased seizure frequency); possible overdiagnosis (e.g., first-ever seizure without other neurological signs, or schizophrenia with a seven-year history and recent behavioral and speech deterioration); newly emerging memory disturbances (as in the MS patient); patient request (borderline personality case); and misdiagnosis (stroke case). After a short follow-up (ranging from a few days to six months), the diagnoses were revised to other chronic conditions (see Table 6), and no additional symptoms suggestive of AE were observed.

Table 7 presents the clinical symptoms, and Figure 2 shows the symptom distribution among AE patients and Ab-positive individuals without AE.

## 3. Discussion

In our retrospective case series, 65 patients (64 adults and 1 adolescent) were evaluated for AE. The majority met criteria for Ab-positive (definite) AE (*n* = 47; 72.3%) or Ab-negative AE (*n* = 7; 10.8%), while an additional group was Ab-positive without meeting AE criteria (*n* = 11; 16.9%). Despite the modest cohort size, we observed complex and heterogeneous clinical features, reflecting both diagnostic challenges and the broad spectrum of autoimmune neurological disorders encountered in practice.

### 3.1. Antibody-Positive AE

Anti-NMDAR AE was the most common subtype among Ab-positive AE patients (>50%), in line with previous studies reporting rates from 30% [15] to over 60% [16,17]. NMDARs are ligand-gated cation channels critical for synaptic transmission and plasticity, widely distributed in the forebrain and limbic system, especially the hippocampus, explaining the disorder’s complex and heterogeneous presentation. Typical features include acute or subacute psychiatric symptoms and memory impairment, often with seizures, speech disturbances, movement disorders, and sleep abnormalities [4,12]. The condition predominantly affects children and young adults (median age: twenties), shows a strong female predominance (4:1), and is frequently associated with tumors (notably ovarian teratomas) and viral encephalitis, particularly herpes simplex encephalitis caused by HSV-1–the most recognized trigger of NMDAR autoimmunity [18,19].

Compared to previous reports, our anti-NMDAR AE patients were demographically distinct, with a notably higher median age at onset (>50 years). This difference is unlikely to result solely from our admission policy, as adult patients are routinely accepted, and younger individuals are only occasionally admitted. It may reflect the characteristics of our population but also suggests that anti-NMDAR AE in older adults is underdiagnosed. One contributing factor may be the presence of multiple adult psychiatric departments in our hospital, facilitating recognition and referral of older patients with prominent neuropsychiatric symptoms. This is relevant, as psychiatric manifestations, particularly first-episode psychosis, often precede neurological signs in adult-onset anti-NMDAR AE [5,6]. Conversely, underdiagnosis in older adults may stem from symptom overlap with non-inflammatory cognitive disorders (e.g., delirium, dementia), leading to fewer diagnostic procedures such as lumbar puncture and antibody testing [5]. The higher prevalence of other AE subtypes in this age group (especially anti-LGI1 and anti-CASPR2), along with fewer tumor associations (e.g., ovarian teratomas), may further reduce suspicion. Atypical or mild presentations may also be overlooked. Notably, in our cohort, most patients who did not meet diagnostic criteria for AE were anti-NMDAR Ab-positive.

Less surprising is the difference in anti-NMDAR AE occurrence between women and men, with a 2:1 ratio. While early studies reported a fourfold female predominance, more recent research indicates reduced sex disparity after the age of 45 [20], and some cohorts have shown an almost equal distribution [21].

Clinically, most of our anti-NMDAR AE patients presented with features resembling LE, though several atypical extralimbic forms were also observed. Rare variants involving cortico-subcortical regions or the brainstem, increasingly recognized in the literature [22,23], were initially misdiagnosed as parkinsonian syndromes or cerebellar disorders in our cohort, leading to diagnostic delays.

Several unusual and diagnostically challenging cases were also identified. Coexistence of anti-NMDAR AE and MS, a difficult-to-recognize overlap due to shared clinical and radiological features, was observed in two patients. Both presented with new-onset epilepsy without prior history. One was a young woman diagnosed simultaneously with MS and AE based on serum and CSF anti-NMDAR Abs and demyelinating MRI lesions. The other had a 20-year MS history and developed non-motor status epilepticus followed by psychosis and cognitive decline. The comorbidity of anti-NMDAR AE and demyelinating syndromes is estimated to occur in approximately 4% of patients with anti-NMDAR encephalitis, with both disorders developing either sequentially or simultaneously [12,20]. Although the pathophysiological relationship remains unclear, a bidirectional link has been proposed: immune activation during AE may disrupt immune regulation and promote demyelination, as NMDARs are expressed on oligodendrocytes and myelin sheaths; conversely, inflammation in demyelinating disease may expose neuronal antigens and trigger secondary anti-NMDAR autoimmunity [24].

We also noted temporal associations between anti-NMDAR AE and both herpes labialis and COVID-19 vaccination. While HSV-1 encephalitis is a well-established trigger of secondary anti-NMDAR AE [25,26], nonencephalitic HSV-1 infections (e.g., oral or genital lesions) are generally considered low risk. However, their potential role cannot be excluded. Proposed mechanisms include HSV-1–induced alterations in NMDAR expression outside the CNS, increasing receptor immunogenicity, or broader immune modulation leading to a breakdown of tolerance and autoimmune targeting of NMDARs [27]. The role of COVID-19 vaccination in triggering anti-NMDAR AE also remains uncertain. Although post-vaccination CNS immune-mediated events are rare and typically Ab-negative [28], isolated cases of anti-NMDAR AE have been reported [29]. In our case, however, the short interval between vaccination and symptom onset suggests a coincidental temporal association rather than a causal link.

Anti-LGI1 and Anti-CASPR2 AE were the second and third most common subtypes in our cohort. Their target antigens, functionally linked to the voltage-gated potassium channel complex, show considerable clinical and radiological overlap. Both primarily affect older adults (median age in the sixties) and are more frequent in men, especially CASPR2 (male-to-female ratio ~9:1 vs. 2:1 for LGI1). Tumor associations are uncommon, usually involving thymomas [4,30].

LGI1 is predominantly expressed in the hippocampus and temporal cortex, explaining its typical LE presentation. Two hallmark features are seizures (particularly FBDS) and short-term memory loss, with psychiatric symptoms and hyponatremia also common [4,30,31,32]. RPD, though rare, can mimic neurodegenerative or prion-like syndromes [33]. One RPD case in our series involved coexisting LGI1 and NMDAR Abs–an extremely rare combination [34] previously described in autoimmune dementia [33]. This highlights the importance of NMDAR Ab testing in atypical or unusually severe LGI1-LE cases.

CASPR2 is an axonal membrane protein expressed in both central and peripheral neurons and has a broader clinical spectrum than LE. Presentations may include peripheral nerve hyperexcitability (e.g., Isaacs’ syndrome with myokymia), combined central–peripheral involvement, isolated pain syndromes, autonomic dysfunction, and occasionally coexisting myasthenia gravis, particularly in thymoma-associated cases [30]. In our cohort, the most common manifestations were LE, RPD, and cerebellitis. The absence of myokymia may reflect predominant CNS involvement, under-recognition of subtle motor signs, or phenotypic variability within CASPR2 autoimmunity.

Anti-GABA_B_R and anti-AMPAR AE were represented by individual cases in our cohort. These rare subtypes typically affect older adults (median age: sixties for anti-GABA_B_R AE; sixties–seventies for anti-AMPAR AE) and usually present as LE, often associated with tumors and coexisting Abs [4,13,30,33]. Among the two anti-GABABR cases, one involved a patient with SCLC and ataxia who was also positive for anti-Hu Abs; the other developed subacute cerebellar symptoms and was later diagnosed with primary progressive MS. In both, overlapping neurological diagnoses (e.g., multiple sclerosis or paraneoplastic syndromes) complicated interpretation, and the pathogenic relevance of detected Abs remained uncertain. The anti-AMPAR case was an elderly woman with RPD, non-small cell lung cancer, and coexisting anti-Hu Abs, consistent with reported patterns.

### 3.2. Antibody-Negative AE

An important aspect of our study is the clinical presentation of Ab-negative AE, a less well-characterized form than Ab-positive AE, diagnosed using established criteria after excluding alternative causes. Although heterogeneous, Ab-negative AE is increasingly recognized as a distinct entity, with presentations ranging from classic LE to acute disseminated encephalomyelitis (ADEM) and atypical or incomplete syndromes [35,36,37]. Many patients show typical limbic MRI changes, inflammatory CSF findings, and respond to immunotherapy, supporting a criteria-based diagnosis and timely treatment despite absent detectable Abs [35,38,39].

In our cohort, seven patients met diagnostic criteria: six had typical LE with poor outcomes (three with persistent symptoms, three deaths), and one had both limbic and extralimbic features and made a full recovery. In the latter, an ovarian teratoma raised suspicion of anti-NMDAR AE, and a false-negative Ab result cannot be excluded due to assay sensitivity limitations [35]. The poorer prognosis of Ab-negative AE compared with Ab-positive AE—particularly anti-NMDAR AE, often largely reversible—has been reported and may reflect heterogeneous pathological mechanisms, including cytotoxic injury [36].

### 3.3. Antibody-Positive, Non-AE Presentations

In addition to confirmed AE cases, our cohort included patients with identified Abs whose clinical features were ultimately attributable to other conditions, highlighting the problem of AE misdiagnosis. Previous reviews report that approximately 25% of presumed AE cases are ultimately reclassified [40,41]. A key contributor is failure to apply diagnostic criteria for probable AE; overinterpretation of non-specific serum Ab results—without a compatible clinical syndrome—accounts for up to 30% of errors. Other causes include attributing functional or non-specific symptoms to AE, overinterpretation of imaging findings, and contextual influences such as reluctance to accept psychiatric diagnoses.

Eleven of our patients (17%)—7 anti-NMDAR and 4 anti-CASPR2—had an initial AE diagnosis later revised to alternative conditions, including epilepsy, multiple sclerosis, stroke, or borderline personality disorder. Excluding alternative diagnoses remains central to accurate AE classification. Some patients ultimately found not to have AE had nonetheless received immunosuppressive therapy.

There is ongoing debate on whether serum positivity without CSF Abs is sufficient for diagnosis. In our series, we believe the serum-positive/CSF-negative patients met established AE criteria, however, low-titer Abs may occasionally occur in healthy individuals without symptoms, complicating interpretation. For GAD65 encephalitis, high Ab titers are considered essential, but for other antineuronal Abs such thresholds remain undefined.

### 3.4. Comparison of Symptom Profiles Across Study Subgroups

Analysis of symptom distribution in Ab-positive AE, Ab-negative AE, and Ab-positive non-AE patients—based on distribution charts and visualization of relationships between clinical symptoms, Abs, and outcomes—revealed distinct patterns. Consistent with previous reports [4,12,30], Ab-positive AE patients exhibited the broadest symptom spectrum, dominated by psychiatric (~25%) and cognitive (~17%) features, but also including seizures, motor deficits, and other neurological signs. This likely reflects the predominance of anti-NMDAR AE with limbic involvement, as well as limbic presentations in anti-LGI1 and anti-CASPR2 AE. Less frequent findings involved the brainstem, corticosubcortical, or cerebellar regions. Certain features provided diagnostic clues, such as speech disturbances in anti-NMDAR and anti-CASPR2 subgroups, and hyponatremia in anti-LGI1 and anti-CASPR2 patients.

Ab-negative AE was characterized by a marked predominance of psychiatric symptoms (~49%), followed by cognitive and consciousness disturbances, and other neurological signs. A similar predominance of psychiatric manifestations has been reported in previous studies, although usually without direct comparison to other AE subtypes [36].

In Ab-positive patients whose symptoms initially suggested AE but in whom the diagnosis was ultimately excluded, seizures (~29%), motor (~24%) and psychiatric manifestations (~19%) predominated, due to conditions such as epilepsy, psychiatric disorders, stroke, or brain tumors.

## 4. Materials and Methods

This single-center, retrospective case series was conducted between 2023 and 2024 at a tertiary referral center for neurological and psychiatric disorders in Warsaw, Poland, serving a broad catchment area, including a densely populated and demographically older urban district (median age ~43 years; 22% aged ≥ 65 [42]). The study was conducted in accordance with the STROBE guidelines [43].

We searched hospital databases using the terms “autoimmune encephalitis”, “limbic encephalitis”, and the ICD-10 code G04 (a broad category encompassing encephalitis, myelitis, and encephalomyelitis not classified elsewhere), cross-referencing them with results from our in-house neuroimmunology laboratory. The search included inpatients from the 1st and 2nd Neurology Departments as well as outpatients, and covered the period from 2013 to 2024.

The initial search identified 367 patients with a G04 diagnosis, the majority with encephalitis of infectious origin (e.g., viral, bacterial, or other) and paraneoplastic encephalitis with Abs to intracell antigens (amphiphisin, CV2, PNMA2, Ri, Yo, recoverin, SOX1, zic4, GAD65, Tr, titin). Of these, 98 were suspected of AE. Each case was independently reviewed by three researchers (K.K., I.K.-J., A.P.) for clinical and paraclinical data (e.g., serum/CSF analysis, brain MRI, EEG), with regard to serological Ab findings and fulfillment of the possible AE criteria as proposed by Graus et al. [12]: (1) subacute onset (<3 months) of memory deficits, altered mental status (decreased/altered consciousness, lethargy, or personality change), or psychiatric symptoms; (2) at least one supportive feature, such as new focal CNS findings, unexplained seizures, CSF pleocytosis > 5 cells/μL, or MRI abnormalities; (3) exclusion of alternative causes.

All reviewers had to agree on the final diagnosis; doubtful cases lacking sufficient data from hospitalization and follow-up were excluded. As a result, 33 patients not meeting the clinical criteria for possible AE and testing negative for Abs in both serum and CSF were excluded, leaving a final cohort of 65 patients. These patients (excluding those who died during hospitalization) were followed in the outpatient clinic for a minimum of 1 year with complete follow-up, to validate the diagnosis and ensure appropriate treatment as part of routine care. Eleven Ab-positive patients who did not meet the Graus criteria at disease onset were retained in the cohort and reviewed for potential misdiagnosis.

**Figure 3:**
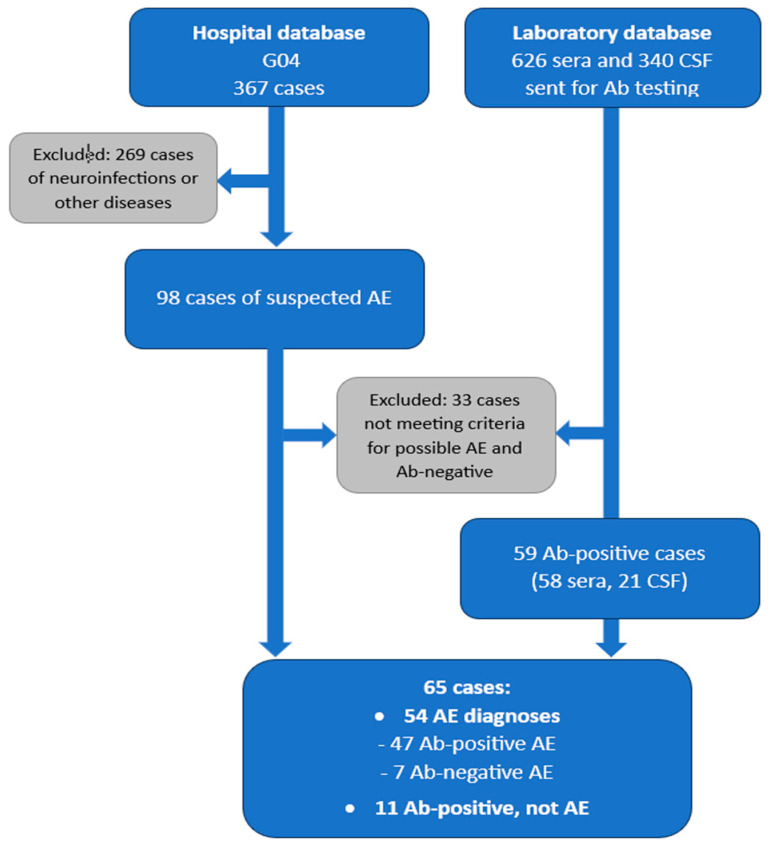
Flowchart illustrating the diagnostic categorization process for patients evaluated for AE. Data were collected from hospital and laboratory databases, with stepwise exclusions based on alternative diagnoses or AE diagnostic criteria, resulting in final categorization into Ab-positive AE, Ab-negative AE, and Ab-positive non-AE cases. Abbreviations: Ab, autoantibody; AE, autoimmune encephalitis; CSF, cerebrospinal fluid.

Integration of clinical diagnosis and Ab status allowed classification of patients into three groups:

- Ab-positive AE: meeting clinical criteria for AE with detectable Abs (*n* = 47);
- Ab-negative AE: meeting clinical criteria for AE without detectable Abs (*n* = 7);
- Ab-positive, not AE: Ab positivity without meeting clinical criteria for AE (*n* = 11) (see Figure 3).

### 4.1. Autoantibody Testing

All tests were performed at the certified Neuroimmunology Laboratory of the Second Department of Neurology, Institute of Psychiatry and Neurology, Warsaw, Poland. Blood and CSF samples were collected simultaneously and processed immediately. Serum and CSF were stored at –80 °C and tested within 7–15 days using EUROIMMUN commercial kits with EU90-transfected cells. From 2014 to 2016, Abs against NMDA, GABA_B_, AMPA1, AMPA2, LGI1, and CASPR2 were detected using Mosaic 1 (FA 112d-1003-1; EUROIMMUN Medizinische Labordiagnostika AG, Lübeck, Germany); from 2017–2022, Abs against NMDA, AMPA_1/2_, GABA_B_, DPPX, LGI1, and CASPR2 were detected using Mosaic 6 (FA 112d-1005-6; EUROIMMUN, Germany). Abs against IgLON5 and GABA_A_ were not tested.

### 4.2. Statistical Analysis

All statistical analyses were performed in Python, version 3.11.9, using the SciPy statistical package (version 1.11.4). Descriptive statistics were used to summarize demographic and clinical variables. Continuous variables were expressed as medians with first and third quartiles (Q1–Q3) due to non-normal distribution confirmed by normality testing. Categorical variables were reported as percentages or ranges. Because of the non-normal distribution of age data, presence of outliers, and small subgroup sizes, a non-parametric Kruskal–Wallis test was used to compare patient ages across Ab-defined groups (anti-NMDAR, anti-LGI1, and anti-CASPR2). As the Kruskal–Wallis test did not yield a statistically significant result, no post hoc pairwise comparisons were performed, and *p*-value correction was not required. One patient positive for both anti-NMDAR and anti-LGI1 Abs was excluded from age comparisons due to phenotypic overlap and uncertain Ab attribution. Patients with anti-GABA_B_R (*n* = 2) and anti-AMPAR (*n* = 1) Abs were excluded due to small sample sizes. A *p*-value < 0.05 was considered statistically significant.

To explore relationships between symptoms and clinical outcomes in Ab-positive AE, Jaccard and Dice dissimilarity coefficients were calculated to construct dissimilarity matrices. These were visualized using t-distributed stochastic neighbor embedding (t-SNE) for Jaccard distances, hierarchical clustering, and heat maps for both Jaccard and Dice distances (Figure 1). Although t-SNE is primarily used for dimensionality reduction, here it represented similarities between features (e.g., symptoms, antibodies), not individual cases. Plots were color-coded according to clusters identified via hierarchical clustering of the same dissimilarity matrices. Due to the limited sample size, reliable similarity results were obtained only for the anti-NMDAR, anti-LGI1, and anti-CASPR2 subgroups, which had the largest number of cases.

## 5. Conclusions

AE is more common than previously recognized and should be considered in the differential diagnosis of both acute and chronic de novo neurological and psychiatric presentations. In addition to well-characterized syndromes such as LE, AE can present with a wide range of neurological symptoms that mimic other disorders and may follow highly variable clinical courses. However, the mere detection of antineural Abs is not sufficient to establish the diagnosis of AE, as such Abs may also be found in other neurological or psychiatric conditions. Diagnosis therefore requires thorough clinical assessment and the exclusion of alternative causes. It is also possible that antineural Abs contribute to transient neurological disturbances without the full development of the AE phenotype (e.g., epilepsy in MS). Careful interpretation is essential, particularly given the rapidly evolving nature of this field.

## 6. Limitation

Our analysis is limited by its retrospective design and the small, single-center sample size. Certain AE subtypes, such as GABA_B_R and AMPA receptor encephalitis, were represented by single cases. We acknowledge that many cases may have been missed, particularly more than a decade ago, when awareness and understanding of AE were limited. In contrast, AE is now more widely recognized and routinely considered in the diagnostic workup of relevant clinical syndromes. To ensure accuracy, we deliberately excluded all uncertain cases and included only those deemed clinically plausible.

## Figures and Tables

**Table 1:** Autoantibody distribution in patients with Ab-positive AE and Ab-positive individuals without AE.

Antibody Profile	Ab-Positive AE (*n* = 47)					Ab-Positive, Not AE (*n* = 11)	
	NMDAR	LGI1	CASPR2	GABA_B_R	AMPAR	NMDAR	CASPR2
Total Abs (N = 59)	*n* = 24	*n* = 13	*n* = 8	*n* = 2	*n* = 1	*n* = 7	*n* = 4
Age: median (Q1–Q3), range [y]	51.5 (38.8–65.2), 22–84	52.0 (40.0–69.0), 16–77	70.0 (59.8–73.8), 21–78	66.0 (65.5–66.5), 65–67	65 *	48.0 (29.0–60.5), 20–66	24,5 (20.5–29.8), 19–35
Serum+ & CSF+	7	7	1	0	0	0	0
Serum+ & CSF–/NT	8/8	2/4	6/1	0/2	1/0	0/7	2/2
Serum– & CSF+	1	0	0	0	0	0	0
Abs in Females (N = 32)	*n* = 16	*n* = 4	*n* = 3	*n* = 2	*n* = 1	*n* = 3	*n* = 3
Age: median (Q1–Q3), range [y]	54.5 (38.2–65.5), 22–77	69.5 (55.0–71.5), 16–73	76.0 (48.5–77.0), 21–78	66.0 (65.5–66.5), 65–67	65 *	65.0 (44.0–65.5), 23–66	28,0 (23.5–31.5), 19–35
Serum+ & CSF+	5	0	0	0	0	0	0
Serum+ & CSF–/NT	3/7	2/2	3/0	0/2	1/0	0/3	2/1
Serum– & CSF+	1	0	0	0	0	0	0
Abs in Males (N = 27)	*n* = 8	*n* = 9	*n* = 5	*n* = 0	*n* = 0	*n* = 4	*n* = 1
Age: median (Q1–Q3), range [y]	44.0 (40.2–55.5), 23–84	50.0 (40.0–66.0), 23–77	67.0 (67.0–73.0), 38–73	–	–	41.5 (31.25–50.0), 20–56	21 *
Serum+ & CSF+	2	7	1	–	–	0	0
Serum+ & CSF–/NT	5/1	0/2	3/1	–	–	0/4	0/1

**Legend.** Abbreviations: Ab, autoantibody; AE, autoimmune encephalitis; CSF−, negative cerebrospinal fluid test; CSF+, positive cerebrospinal fluid test; Q1, first quartile; Q3, third quartile; N, total number in the category; *n*, number in the subgroup; NT, not tested; serum−, negative serum test; serum+, positive serum test; y, years. A dash (–) denotes the absence of cases with the respective Abs in the given subgroup. An asterisk (*) denotes subgroups represented by a single patient, for which age is reported as an individual value.

**Table 2:** Cerebrospinal fluid findings in patients with AE and Ab-positive individuals without AE.

Cerebrospinal Fluid	Ab-Positive AE			Ab-Negative AE			Ab-Positive, Not AE		
	Total (N = 47)	Female (*n* = 26)	Male (*n* = 21)	Total (N = 7)	Female (*n* = 3)	Male (*n* = 4)	Total (N = 11)	Female (*n* = 6)	Male (*n* = 5)
Pleocytosis (cells/μL)									
0–5 (NR)	29	18	11	5	3	2	8	6	2
6–50	9	3	6	2	–	2	–	–	–
>50	3	3	–	–	–	–	–	–	–
NT	6	2	4	–	–	–	3	–	3
Protein (mg/dL)									
<45 (NR)	21	12	9	4	2	2	4	3	1
45–100	13	5	8	3	1	2	2	1	1
>100	–	–	–	–	–	–	–	–	–
NT	13	9	4	–	–	–	5	2	3
IgG Index									
<0.7 (N)	30	19	11	7	3	4	7	5	2
>0.7	10	5	5	–	–	–	–	–	–
NT	7	2	5	–	–	–	4	1	3
Oligoclonal Bands									
Type I	14	10	4	4	3	1	2	1	1
Type II	6	3	3	–	–	–	–	–	–
Type III	4	2	2	–	–	–	2	2	–
Type IV	6	1	5	–	–	–	–	–	–
Type V	1	1	–	–	–	–	–	–	–
NT	16	9	7	3	–	3	7	3	4

**Legend.** Ab, autoantibody; AE, autoimmune encephalitis; N, total number in the category; *n*, number in the subgroup; NR, normal range based on institutional laboratory reference values; NT, not tested. A dash (–) denotes no cases with such results in the respective subgroup.

**Table 3:** MRI findings in patients with AE and Ab-positive individuals without AE.

MRI Findings	Ab-Positive AE (N = 47)		Ab-Negative AE (N = 7)	Ab Positive, Not AE (N = 11)	
	*n*	Associated Ab	*n*	*n*	Associated Ab
No abnormalities detected	14	anti-NMDAR (*n* = 7) anti-CASPR2 (*n* = 3) anti-LGI1 (*n* = 2) anti-GABA_B_R (*n* = 1) anti-NMDAR/LGI1 (*n* = 1)	1	5	anti-NMDAR (*n* = 4) anti-CASPR2 (*n* = 1)
Vasogenic edema/injury	16	anti-NMDAR (*n* = 8) anti-CASPR2 (*n* = 4) anti-LGI1 (*n* = 3) anti-AMPAR/Hu (*n* = 1)	1	3	anti-CASPR2 (*n* = 2) anti-NMDAR (*n* = 1)
Limbic inflammation	14	anti-LGI1 (*n* = 7) anti-NMDAR (*n* = 6) anti-GABA_B_R (*n* = 1)	5	–	–
Demyelinating changes	2	anti-NMDAR (*n* = 2)	–	1	anti-NMDAR (*n* = 1)
Neoplastic changes	–	–	–	2	anti-NMDAR (*n* = 1) anti-CASPR2 (*n* = 1)

**Legend.** Abbreviations: Ab, autoantibody; AE, autoimmune encephalitis; N, total number in the category; *n*, number in the subgroup. A dash (–) denotes no cases with such findings in the respective subgroup.

**Table 4:** Neoplasm occurrence in patients with AE and Ab-positive individuals without AE.

Neoplasm Distribution	Ab-Positive AE (N = 47)		Ab-Negative AE (N = 7)	Ab-Positive, Not AE (N = 11)	
	*n*	Associated Ab	*n*	*n*	Associated Ab
Ovarian tumor: carcinoma/teratoma	2:1/1	anti-NMDAR	1:0/1	–	–
Renal cell carcinoma	1	anti-NMDAR	–	–	–
Nasopharynx carcinoma	1	anti-LGI1	–	–	–
Breast carcinoma	1	anti-NMDAR	–	–	–
Non-small cell lung cancer	1	anti-AMPAR/Hu	–	1	anti-NMDAR
Small cell lung cancer	1	anti-GABA_B_R	–	–	–
Myeloma	2	anti-NMDAR anti-CASRP2	–	–	–
Melanoma	1	anti-LGI1	–	–	–
Brain neoplasm	–	–	–	2	anti-NMDAR anti-CASPR2
Not detected/Unknown	30/7	–	–	8/1	–

**Legend.** Abbreviations: Ab, autoantibody; AE, autoimmune encephalitis; N, total number in the category; *n*, number in the subgroup. A dash (–) denotes no cases with such conditions in the respective subgroup.

**Table 5:** Immunosuppressive treatment in patients with AE and Ab-positive individuals without AE.

Treatment Modality	Details	AE (N = 54)	Ab-Positive, Not AE (N = 11)
		*n*	*n*
Methylprednisolone (MP)	5 × 1 g IV	33	4
Intravenous Immunoglobulin (IVIG)	2 g/kg	26	2
Plasma Exchange (PLEX)	5–7 sessions, every other day	10	–
Cyclophosphamide (CYC)	1000 mg, 3–5 cycles every 4 weeks	11	–
Rituximab	As per protocol	1	–
Prolonged immunosuppressive treatment	1–5 years	28	3
Oral steroids	Prednisone 1 mg/kg, 3–24 months	16	3
Other immunosuppressant	Azathioprine	7	1
	Mycophenolate mofetil	1	–
	Methotrexate	4	–
No treatment	—	7	5

**Legend.** Abbreviations: Ab, autoantibody; AE, autoimmune encephalitis; N, total number in the category; *n,* number in the subgroup. The dash (–) denotes that no patients received the treatment in the respective subgroup.

**Table 6:** Revisions of diagnosis in Ab-positive patients targeting NMDAR and CASPR2.

Autoantibody	*n*	Sex/Age	Initial Symptoms	Final Diagnosis
Anti-NMDAR (N = 7)	1	F/28	first seizure	primary generalized epilepsy
	3	F/23, M/27, M/56	first seizure	epilepsy of unknown etiology
	1	M/35	initial psychotic episode	catatonic schizophrenia
	1	F/65	limb paresis, ataxia, mild memory impairment	primary progressive MS
	1	F/66	hemiparesis, dyskinesia, altered consciousness	ischemic stroke
Anti-CASPR2 (N = 4)	2	F/35, M/48	first seizure	glioma (2 cases)
	1	M/21	first seizure	epilepsy of unknown etiology
	1	F/19	behavioral disturbances	borderline personality disorder

**Legend.** Abbreviations: F, female; M, male; N, total number in the category; *n*, number in the subgroup.

**Table 7:** Clinical symptoms in AE patients and Ab-positive individuals without AE.

Clinical Characteristic	Ab-Positive AE			Ab-Negative AE			Ab-Positive, Not AE		
	Total (N = 54)	Female (*n* = 29)	Male (*n* = 25)	Total (N = 7)	Female (*n* = 3)	Male (*n* = 4)	Total (N = 11)	Female (*n* = 6)	Male (*n* = 5)
Age, median (Q1–Q3), range [y]	61.0 (41.0–68.5), 16–84	64.5 (41.25–70.25), 16–78	52.0 (42.0–67.0), 23–84	55.0 (38.5–64.0), 26–80	55.0 (40.5–62.0), 26–69	50.0 (39.8–64.3), 36–80	35.0 (22.0–52.0), 19–66	31.5 (24.2–57.5), 19–66	35.0 (21.0–48.0), 20–56
*Symptoms*									
Altered consciousness	18	11	7	5	3	2	2	1	1
Behavioral (psychiatric)	22	14	8	6	2	4	1	1	–
Psychotic	10	7	3	8	2	6	2	1	1
Anxiety/Depression/Bipolar disorder	4/2/1	3/2/1	1/0/0	4/1/0	1/0/0	3/1/0	1/0/0	1/0/0	–
Neuroleptic malignant syndrome	2	1	1	2	0	2	–	–	–
Catatonia	2	1	1	–	–	–	–	–	–
Memory disorders/Dementia	20/9	9/4	11/5	6/0	2/0	4/0	1/0	0	1/0
Seizures/Status epilepticus/Myoclonus	16/7/1	7/5/1	9/2/0	1/1/0	1/1/0	0	6/0/0	3/0/0	3/0/0
Speech disorders	10	7	3	3	3	0	–	–	–
Parkinson’s syndrome/Dyskinesia/PSP-like	4/7/1	1/4/1	3/3/0	–	–	–	0/1/0	0/1/0	–
Lower limb paresis	5	3	2	–	–	–	–	–	–
Pyramidal syndrome	1	1	0	–	–	–	1	1	–
Focal symptoms	3	2	1	1	0	1	2	2	–
Cerebellar syndrome	4	3	1	1	1	0	–	–	–
Brainstem: oculomotor/urinary/dysphagia	1/4/1	1/3/1	0/1/0	1/0/0	1/0/0	0/0/0	1/0/0	1/0/0	–
Autonomic dysfunction	4	3	1	–	–	–	–	–	–
Sleep disorders	4	4	0	2	0	2	3	2	1
Pain/Paresthesia	2/1	0/1	2/0	–	–	–	–	–	–
Polyneuropathy	1	0	1	–	–	–	–	–	–
Hyponatremia	5	5	0	–	–	–	–	–	–
Weight loss	1	1	0	1	0	1	–	–	–
*Outcome*									
Complete recovery	3	2	1	1	1	0	1	1	–
One-time relapse	4	3	1	–	–	–	–	–	–
Epilepsy	16	5	11	1	0	1	6	3	3
Memory disorders	15	7	8	2	0	2		–	–
Psychiatric symptoms	9	3	6	1	1	0	3	2	1
Multiple sclerosis	2	2	0	–	–	–	1	1	–
Death	14	11	3	3	1	2	1	–	1
*Final diagnosis*									
LE	34	20	14	7	3	4	–	–	–
RPD	6	2	4	–	–	–	–	–	–
Brainstem AE	3	2	1	–	–	–	–	–	–
Cortico-subcortical AE	2	1	1	–	–	–	–	–	–
Cerebellitis AE	2	1	1	–	–	–	–	–	–
Epilepsy	–	–	–	–	–	–	5	2	3
Glioma	–	–	–	–	–	–	2	1	1
Stroke	–	–	–	–	–	–	1	1	0
MS	–	–	–	–	–	–	1	1	0
Schizophrenia	–	–	–	–	–	–	1	0	1
Borderline personality	–	–	–	–	–	–	1	1	0

**Legend.** Abbreviations: Ab, autoantibody; AE, autoimmune encephalitis; Q1, first quartile; Q3, third quartile; N, total number in the category; *n*, number in the subgroup; y, years. The dash (–) denotes no cases with such symptoms in the respective subgroup. Values separated by slashes indicate counts for subcategories as listed in the table row.

## Data Availability

The data presented in this study are available on request from the corresponding author. The original contributions presented in this study, consisting of anonymized patient data (Excel file), are available upon reasonable request from the corresponding author, Agnieszka Piechal; further inquiries can also be directed to Iwona Kurkowska-Jastrzębska.

## Abbreviations

Ab	autoantibody (antibody)
AE	autoimmune encephalitis
AMPAR	α-Amino-3-hydroxy-5-methyl-4-isoxazole-propionic acid receptor
CASPR2	contactin-associated protein-like 2
CBA	cell-based assay
CSF	cerebrospinal fluid
FBDS	faciobrachial dystonic seizures
GABA_B_R	γ-Aminobutyric acid receptor-B
HSV-1	herpes simplex virus 1
IVIG	intravenous immunoglobulin
LE	limbic encephalitis
LGI1	components of the voltage-gated potassium channel complex, including leucine-rich glioma-inactivated protein 1
MP	methylprednisolone
MS	multiple sclerosis
NMDAR	N-methyl-D-aspartate receptor
PLEX	plasma exchange
RPD	rapidly progressive dementia
SCLC	a small-cell lung carcinoma
